# Pediatric motor vehicle crashes injuries: A systematic review for forensic evaluation

**DOI:** 10.1007/s00414-024-03174-7

**Published:** 2024-02-10

**Authors:** Elena Giovannini, Simone Santelli, Guido Pelletti, Maria Paola Bonasoni, Angela Cornacchia, Susi Pelotti, Paolo Fais

**Affiliations:** Department of Medical and Surgical Sciences, Unit of Legal Medicine, University of Bologna, Via Irnerio 49, Bologna, 40126 Italy

**Keywords:** Pediatric injuries, Forensic pathology, Accident dynamics, Restraint system, Traffic accidents

## Abstract

Children involved in car crashes can experience either direct trauma or inertial injuries resulting from interactions with external objects, such as other vehicles, or with the restraint system. Furthermore, improper use of restraint systems can lead to additional severe injuries. Recent reports from international institutions underscored the persistent prevalence of inadequate restraint systems utilization and this widespread issue increases children’s vulnerability and risk of injuries.

The aim of this study is to provide a systematic review of the literature on injuries sustained in children involved in road accidents describing and analyzing elements useful for forensic assessment.

The literature search was performed using PubMed, Scopus and Web of Science from January 1970 to March 2023. Eligible studies have investigated issues of interest to forensic medicine about traffic accidents involving pediatric passengers. A total of 69 studies satisfied the inclusion criteria and were categorized and analyzed according to the anatomical regions of the body affected (head, neck, thoraco-abdominal, and limb injuries), and the assessment of lesions in reconstruction of the accident was examined and discussed.

The review highlights that in motor vehicle accidents involving children, the forensic evaluation of both the cause of death and accident dynamics needs to consider several factors, such as the child’s age, the type of restraint system employed, and the specific passenger seat occupied. Considering the complexity of the factors that can be involved in this road accident, it is crucial that there is a comprehensive exchange of information between the judge and the medical expert.

## Introduction

Road traffic accidents represent a significant public health challenge, imposing substantial economic and social burdens. Despite a reduction in road-related fatalities in developed countries, these accidents continue to contribute to pediatric mortality and morbidities globally [1–4].

Children involved in car crashes can experience either direct trauma or inertial injuries resulting from interactions with external objects, such as other vehicles, or with the restraint system [5, 6]. Furthermore, improper use of restraint systems can lead to additional severe injuries [7, 8].

The use of restraints, such as car seats and seat belts, must consider the evolving growth and development of the body with age. Infants should be securely buckled in rear-facing car seats, while children aged 9 to 12 no longer require car seats if the seat belt fits them properly and can be worn safely [9–11].

Recent reports from international institutions, including the Italian National Institute of Health, the European Road Safety Authority, the National Safety Council Observatory Report, and the Centers for Disease Control and Prevention, have underscored the persistent prevalence of inadequate restraint systems utilization. This widespread issue remains a significant problem, increasing children’s vulnerability and risk of injuries [12–15].

The complexities inherent in accident dynamics make their reconstruction a challenging task. Typically, the reconstruction of a traffic crash involves interviewing the individuals involved or eyewitnesses, conducting mechanical or engineering examinations, and obtaining recorded images of the accident from digital video sources or event data recorders, but also injuries observed can show distinct and specific characteristics depending on the dynamics of the traffic accident [16–18].

A systematic review of studies reporting injuries observed in children involved in motor vehicle accidents was conducted with the aim of describing and analyzing elements that are useful for forensic assessment. This includes features of reported injuries, their role in causing death, and their relevance in reconstructing the dynamics of the accidents.

## Materials and methods

An electronic search was performed in 3 databases: PubMed, Scopus, and Web of Science. Keywords related to the study aim and included in the search string were: (car OR motor vehicle) AND (traffic accident OR road traffic OR crash OR prevention) AND (injury OR wound) AND (child OR infant OR pediatric). The Preferred Reporting Items for Systematic reviews and Meta-analyses (PRISMA) guidelines were used [19].

The English language and time interval of publication, from January 1970 to March 2023, were applied as filters. All studies that investigate the characteristics of injuries resulting from motor vehicle crashes involving pediatric passengers were included. The following studies were included: (a) studies carried out in the forensic field which involved autopsy of victims; (b) studies performed in clinical settings, involving both living and deceased subjects, from which information on the distribution and production characteristics of injuries could be obtained (c) traffic engineering and public health studies, analysing strategies and technologies for injury prevention in road traffic accidents involving children.

This review did not extract diagnostic or therapeutic implications from the various types of injuries examined.

Titles, abstracts, and full texts were screened for inclusion criteria and examined. References of the selected articles were further screened, and related papers were included as a source of additional data. The following details were collected: authors’ names, article titles, journal names, publication years, article types (such as prospective or retrospective studies, case reports, or original articles), the number of cases, whether the autopsy had been performed and injury localization and mechanism of production.

## Results

**Fig. 1:**
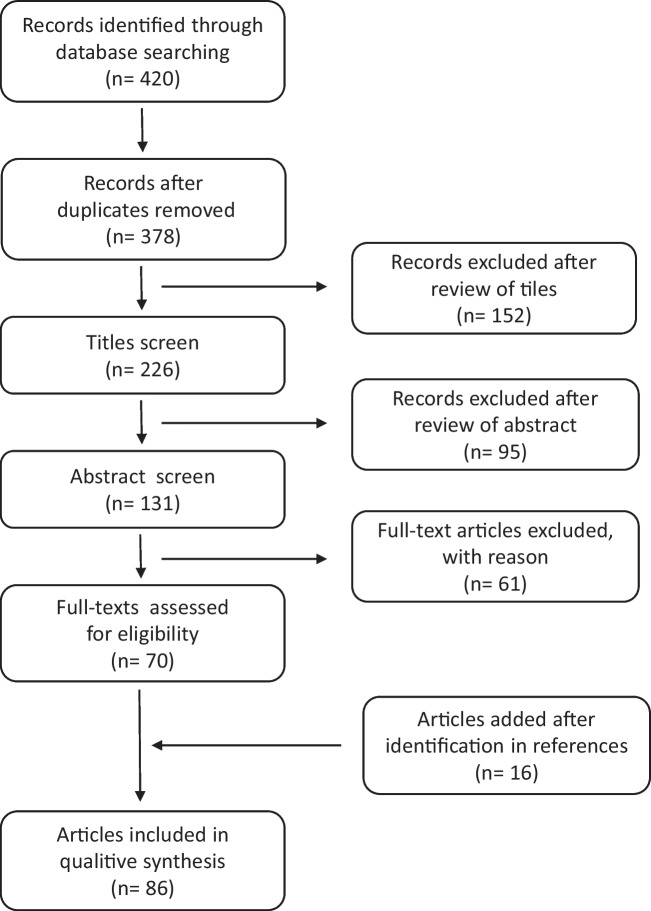
PRISMA flowchart of the present review

The results of the literature search are summarized in Figure 1. Eighty-six studies met the inclusion criteria and were included in the review. The specific characteristics of each study are summarized in Table 1.

**Table 1:** Studies included in the present review

Author	Year	Study type	Number of cases	Autopsy performed	Article issue
Dajee et al [20]	1979	Case report	1	Yes	Abdomen injuries
Newman et al. [21]	1990	Retrospective study	10	No	Abdomen injuries
Johnson et al. [22]	1990	Retrospective study	9	No	Abdomen injuries
Sivit et al. [23]	1991	Retrospective study	61	No	Abdomen injuries
Statter et al. [24]	1992	Case report	1	Yes	Abdomen injuries
Tso et al. [25]	1993	Retrospective study	42	No	Abdomen injuries
Sturm et al. [26]	1995	Retrospective study	7	Yes	Abdomen injuries
Centers et al. [27]	1995	Case report	3	Yes	Head and limbs injuries
Kimmona et al. [28]	1996	Case report	1	Yes	Abdomen injuries
Smith et al. [29]	1997	Case report	2	Yes	Abdomen injuries
Levine et al. [30]	1998	Case report	1	Yes	Abdomen injuries
Cooper et al. [31]	1998	Case report	1	Yes	Head, neck, chest, and abdomen injuries
Marshall et al. [32]	1998	Retrospective study	60	No	Head and neck injuries
Giguère et al. [33]	1998	Retrospective study	30	No	Head and neck injuries
Morrison et al. [34]	1998	Case report	1	Yes	Head and neck injuries
DeCou et al. [35]	1999	Case report	1	Yes	Abdomen injuries
Tyroch et al. [36]	2000	Retrospective study	586	No	Head, neck, chest, abdomen, and limbs injuries
Durbin et al. [37]	2001	Case report	1	No	Abdomen injuries
Uemura et al. [38]	2001	Case report	1	Yes	Neck injuries
Lapner et al. [39]	2001	Retrospective study	45	No	Head, neck, chest, and abdomen injuries
Durbin et al. [40]	2002	Original article	/	No	Head, chest, and limbs injuries
Nichol et al. [41]	2002	Case report	1	No	Chest and abdomen injuries
Arbogaa et al. [8]	2002	Retrospective study	92	No	Head injuries
Bockholdt et al. [42]	2003	Retrospective study	10	No	Head, neck, chest, and abdomen injuries
Orzechowski et al. [43]	2003	Retrospective study	186	No	Head, neck, chest, and abdomen injuries
Ebel et al. [44]	2003	Retrospective study	2880	No	Injury prevention strategies and technologies
Winston et al. [1]	2004	Retrospective study	10195	No	Neck, chest, and abdomen injuries
Prince et al [45]	2004	Case report	4	No	Neck, chest, and abdomen injuries
Davies et al. [46]	2004	Original article	/	No	Head, neck, chest, and abdomen injuries
Quiñones et al. [7]	2005	Retrospective study	263	No	Head, neck, chest, and abdomen injuries
Durbin et al. [47]	2005	Retrospective study	17980	No	Injury prevention strategies and technologies
Santschi et al. [48]	2005	Retrospective study	8	No	Chest and abdomen injuries
Centers et al. [49]	2005	Retrospective study	168	No	Head and limbs injuries
Newgard et al. [50]	2005	Retrospective study	60	No	Head, chest, and abdomen injuries
Arbogast et al. [51]	2005	Retrospective study	1781	No	Injury prevention strategies and technologies
Brown et al. [52]	2006	Retrospective study	417	No	Head, neck, chest, and abdomen injuries
Elliott et al. [53]	2006	Retrospective study	7813	No	Injury prevention strategies and technologies
Papavasiliou et al. [54]	2007	Case report	1	No	Head, chest, and abdomen injuries
Achildi et al. [55]	2007	Retrospective study	121	No	Neck, chest, and abdomen injuries
Jermakian et al. [56]	2007	Retrospective study	20	No	Limbs injuries
Zaloshnja et al. [57]	2007	Retrospective study	409	No	Injury prevention strategies and technologies
Arbogast et al. [58]	2007	Retrospective study	348	No	Injury prevention strategies and technologies
Maltese et al. [59]	2007	Retrospective study	50	No	Head, chest, and abdomen injuries
Lennon et al. [60]	2007	Retrospective study	30631	No	Injury prevention strategies and technologies
Santschi et al. [61]	2008	Retrospective study	28	No	Chest and abdomen injuries
García et al. [62]	2008	Retrospective study	6680	No	Head, chest, and abdomen injuries
O’Neil et al. [63]	2009	Retrospective study	2287	No	Injury prevention strategies and technologies
Clarke et al. [64]	2009	Retrospective study	330	No	Head, neck, and limbs injuries
Brown et al. [65]	2009	Retrospective study	58	No	Head, neck, chest, and abdomen injuries
Arbogast et al. [66]	2009	Retrospective study	314968	No	Injury prevention strategies and technologies
Arbogast et al. [67]	2010	Retrospective study	41	No	Head, neck, chest, and abdomen injuries
Soundappan et al. [68]	2010	Retrospective study	271	No	Head, neck, chest, abdomen, and limbs injuries
Barraco et al. [69]	2010	Retrospective study	/	No	Injury prevention strategies and technologies
Skjerven et al. [70]	2011	Retrospective study	27	No	Head, neck injuries
Toro et al. [71]	2011	Retrospective study	107	No	Head, chest, abdomen, and limbs injuries
Arbogast et al. [9]	2012	Retrospective study	21	No	Head injuries
Andersson et al. [72]	2012	Retrospective study	/	No	Injury prevention strategies and technologies
Moremen et al. [73]	2013	Case report	3	No	Abdomen injuries
Charyk et al. [74]	2013	Retrospective study	106	No	Head, neck, chest and abdomen injuries
Viklund et al. [75]	2013	Retrospective study	20	Yes	Head injuries
Belwadi et al. [76]	2014	Retrospective study	42	No	Head, neck, chest, abdomen, and limbs injuries
Zonfrillo et al. [77]	2014	Retrospective study	42	No	Neck, chest, and abdomen injuries
Stewart et al. [78]	2014	Retrospective study	119	No	Head injuries
Papazoglou et al. [79]	2015	Case report	1	No	Abdomen and limbs injuries
Terranova et al. [80]	2015	Case report	2	Yes	Head and neck injuries
Parrish et al. [81]	2015	Case report	1	No	Neck, chest, and abdomen injuries
Yunus et al. [82]	2015	Retrospective study	19	No	Head injuries
Mitchell et al. [83]	2015	Retrospective study	470	No	Head, chest, and abdomen injuries
Gielen et al. [84]	2015	Retrospective study	/	No	Injury prevention strategies and technologies
Weatherwax et al. [85]	2016	Retrospective study	/	No	Injury prevention strategies and technologies
Meral et al. [86]	2017	Prospective study	746	No	Head, neck, chest, abdomen, and limbs injuries
O’Donovan et al. [87]	2018	Original article	/	No	Head, neck, chest, and abdomen injuries
Davis et al. [88]	2018	Retrospective study	/	No	Head, neck, chest, and abdomen injuries
Takahashi et al. [3]	2018	Retrospective study	371	No	Head, chest, and abdomen injuries
Savenkova et al. [89]	2019	Retrospective study	/	No	Head, chest, abdomen, and limbs injuries
Mallory et al. [90]	2019	Retrospective study	98	No	Neck, chest, and abdomen injuries
Belwadi et al. [91]	2019	Retrospective study	/	No	Injury prevention strategies and technologies
Alghnam et al [92]	2020	Retrospective study	190	No	Head injuries
Jammeh et al. [93]	2020	Case report	4	No	Abdomen injuries
Bohman et al. [94]	2020	Retrospective study	/	No	Injury prevention strategies and technologies
Sarwahi et al. [95]	2021	Sperimental study	34563	No	Head, neck, chest, and abdomen injuries
Laureano et al. [96]	2021	Retrospective study	85	No	Head injuries
Riches et al. [97]	2022	Case report	2	Yes	Neck, chest, and abdomen injuries
Parab et al. [98]	2022	Retrospective study	40	No	Injury prevention strategies and technologies
Spering et al. [99]	2022	Retrospective study	727	No	Head, chest, abdomen, and limbs injuries

A total of 69 studies in the clinical and forensic fields were included in the analysis. Among these, 44 (64%) were retrospective studies, 20 (29%) case reports, 1 (1%) prospective study, and 1 (1%) experimental study. The forensic studies, in which autopsies were carried out, were 20%. The clinical studies, conducted primarily on both living and deceased patients excluding autopsies, were 80% and included the largest number of cases. Abdomen injuries were the most extensively analyzed, appearing in the largest number of studies (50/69), followed by lesions to head (43/69), neck (32/69), chest (37/69), and limbs (13/69). The description of injury features was extracted from each article and thoroughly discussed.

A total of 17 retrospective studies addressing injury prevention strategies and technologies were included.

## Discussion

In motor vehicle accidents involving children, injuries can occur in different parts of the body through different mechanisms. Depending on their severity, these injuries may result in the death of the victim and may provide important insights into the dynamics of the accident. The first four paragraphs discuss the characteristics of the injuries, based on a literature review, categorised by the body regions affected (head, neck, thorax-abdomen, and limbs), and the fifth paragraph discusses the evaluation of injury prevention tests and technologies. Given the broad timespan of the literature review (1970–2023), each paragraph shows the chronological arrangement of the cited sources corresponding to the respective topics under discussion.

The final section examines elements critical to forensic assessment, including the role of injuries in causing fatalities and their importance in reconstructing accident dynamics.

### Head injuries

Head injuries are identified as the most frequent and fatal consequences of pediatric motor vehicle crashes [3, 7, 9, 36, 52, 59, 67, 70, 76, 78, 92]. The prevailing intracranial lesions observed include cerebral contusions, diffuse axonal damage, subdural or subarachnoid haemorrhage, and basilar skull fracture [59, 76]. Infants are vulnerable to sudden head movements as meningeal bridging veins can be easily stretched and torn leading to subdural haemorrhages. The rupture of those vessels can occur even with minor shaking without substantial contact with any structure or another occupant. On the contrary, in older children, head lesions are often associated with skull fractures and other points of impact within the vehicle [9]. Despite proper restraint usage, children involved in motor vehicle collisions often sustain significant head injuries, and the nature of the trauma reflect the type of restraint [78]. For infants, forward-facing car seat are associated with a higher incidence of skull fractures and intracranial damage [9]. Conversely, rear-facing car safety seats offer optimal support to the head and spine during a crash, as forces are directed from the car safety seat’s back to the infant’s back, which represents their strongest body surface [100]. In older children, typically seated in forward-facing car seat, serious facial or scalp lacerations are more common if lap and shoulder belts were used [9].

The improper use of child car seat systems in the front seat, particularly combined with airbags, favours specific injury patterns, explained by the mechanisms associated with airbag trauma [7]. In the case of infants secured in rear-facing car seat systems in the front seat, the airbag strikes the child restraint system and continues to expand, crushing the child’s head. For older children, braking before impact causes them to shift forward, placing them closer to the dashboard. When the airbag deploys, it impacts the face and frontal cranium, inducing violent hyperextension of the cervical spine and subsequent lesions [32]. The lower body mass of children renders them more vulnerable to minor shifts in body position during airbag deployment, resulting in face thermal lesions, abrasions, facial bone fractures, and, in extreme cases, occipitoatlantoaxial disarticulation [31, 34, 39].

In children, irrespective of the seating position inside the vehicle, head and brain injuries are often linked to the inappropriate use of loosely fitting seat belts, as increased head excursion is favoured by inadequate torso restraint. These lesions occur when the child’s head strikes an object in front of her/him, such as the instrument panel without an airbag or the back of the front seats in rear-seated children. The appropriate use of child restraints can help mitigate such injuries by improving torso support and reducing head excursions [8].

In relation to the child’s position within the vehicle during a crash, head damages are more severe in lateral-impact crashes compared to frontal collisions [52]. Lateral impacts can occur between vehicles, fixed objects, such as a wall or road signs, or in rollovers, and often result in severe and potentially lethal craniocerebral injuries [9]. Children are particularly at risk due to their larger head surface area and lower seated height, likely striking the interior door panel or pillars during side-impact crashes [43].

Concerning the temporal distribution of the articles discussed in this paragraph, those addressing the anatomical features of head injuries have all been published from 2000 onwards [3, 7, 9, 36, 52, 59, 67, 70, 76, 78, 92]. Specifically, articles examining head injuries in relation to the child’s seating position and type of crash impacts date back to 10 years ago [9, 52, 78]. Head injuries resulting from the direct impact of airbags have been documented in articles from both the 1990s [31, 32, 34] and the 2000s [7, 39]. Interestingly, there is no mention of such injuries in articles published within the last two decades.

### Neck injuries

In infancy, the cervical spine is the neck region most frequently involved in motor vehicle accidents. Children are particularly vulnerable to cervical spine traumatisms in frontal crashes, in which spine hyperflexion distraction may lead to fractures of cervical vertebrae and brainstem damage [52, 80]. On the other hand, rear impacts can cause whiplash injuries due to hyperextension of the neck and secondary lesions from rebound flexion of the head [5]. Pediatric cervical lesions are more common than in adults due to anatomical differences and distinct damage mechanisms. Infants have a relatively wider and heavier head compared to their body, resulting in a higher centre of gravity and fulcrum of movement in the neck. Therefore, the cause of cervical trauma is rapid acceleration and deceleration. Additionally, centres of vertebral ossification and ligamentous structures are laxer and more fragile in children compared to adults [4]. It has been hypothesized that the location and nature of spinal injuries in children shift as a result of anatomical development during the transition from childhood to adolescence. At children age, the lesions tend to occur at inferior vertebral levels, typically at thoracic location [90].

The correct use of children restraint systems does not fully avoid cervical spine traumas. If the child retained in the car seat is seated in the front passenger seat, airbag deployment during an accident can cause hyperextension of the cervical spine due to facial impact [34, 39, 80]. The action of the shoulder belt during the deceleration phenomena can also damage neck structures, which can be directly hurt, resulting in an oblique abrasion corresponding to the position of the shoulder belt, possibly associated with the rupture of the sternocleidomastoid muscle. In severe trauma, tearing of the common carotid artery, below the sternocleidomastoid muscle, can occur as hyperextension and flexion of the neck compressing the vessel between the seat belt and the vertebral column [97, 101]. Although rare, seatbelt action can transect the trachea [38].

In line with the structure of the paragraph discussing head injuries, articles addressing the anatomical features of head injuries have all been published from 2000 onwards [4, 5, 52, 80, 90]. Neck injuries resulting from the direct impact of airbags have been documented in articles from both the 1990s [31, 32, 34] and the 2000s [7, 39]. Thus, also in this type of injuries, there is no mention of such injuries in articles published within the last two decades.

### Chest and abdomen injuries

Thoracoabdominal injuries, such as cardiac, pulmonary, splenic or hepatic ruptures, aortic transection, and spine, ribs, or clavicle fractures, are less frequent compared to other body regions. They are usually caused by impact with motor vehicle structures, like protuberances on the door interior [59, 71].

Specifically, children involved in lateral-impact collisions often suffer severe thorax trauma compared to frontal crashes, with a much higher fatal outcomes for those seated on the impact side [9, 52].

Furthermore, the movement of the seat belt during impact can result in a distinct injury pattern, and children are especially vulnerable due to their physical and behavioural characteristics. Children’s abdominal wall and musculature are less developed than adults, and the costal margin does not extend as far down, and even minor trauma can lacerate the spleen and liver.

During deceleration, a child’s body moves rapidly forward, and the immature pelvis cannot properly anchor the lap portion of the belt. Additionally, children tend to slide forward in the seat, flexing their knees at the seat edge, allowing the lap belt to override the anterior superior iliac spines and ride up over the abdomen. Consequently, the child’s upper torso hyperflexes over the lap belt, inducing a sudden increase in internal pressure due to a direct compression between the belt and the spine favouring organ contusions and lacerations. These are easily produced as children have a smaller anteroposterior diameter. Similarly, the thoracic cage of children is more flexible than adults, and compression can easily result in severe internal injuries not necessarily damaging bony structures [37, 40, 45, 102, 103].

Lesions associated with the three-point belt, known as lap-shoulder belt, often involve the chest, with fractures of the sternum, ribs, and clavicle, in addition to lesions to the heart, lungs, brachial plexus, and aorta [104].

A distinctive pattern of injury is associated with the two-point belt, known as lap belt, which was first noted by Garrett and Braunstein and termed the seat belt syndrome [45]. Blunt trauma can cause a traumatic abdominal wall hernia with disruption of muscle and fascia, even without penetration of the skin [30, 73].

Solid viscera, namely liver, pancreas, and the spleen, can be subjected to lacerations and perforations may occur in hollow structures, such as the intestine and stomach [24, 37, 93, 97].

The jejunum is the most common site of small intestine involvement, especially around the ligament of Treitz, where fixation promotes shear stress tears. Another vulnerable fixed site is the ileocecal valve [105].

Although rare, seat belt-related abdominal blunt trauma can lead to aortic injuries such as dissection, rupture, thrombosis, or intimal tears [20, 93, 97]. These lesions are related to forces crushing the vessel between the vertebral column and the seat belt. Additionally, elongation of the aorta from the pressure of the decelerating column of blood and changes in intraluminal pressure may exacerbate the damage [106].

In cases of severe trauma, spine fractures or dislocations can be observed, particularly involving the first and second lumbar vertebrae [21, 37, 54, 93, 97]. Chance fractures, a type of compression injury to the lumbar spine, can also be detected. These fractures consist of rupture of the posterior ligaments and fractures of the spinous process, pedicles, and vertebral bodies. The unique characteristic of the Chance fracture is that the fracture line extends transversely through the spinous process, laminae, transverse processes, pedicles, and into the superior surface of the vertebral body [26, 107].

Concerning the temporal distribution of the articles discussed in this paragraph, all articles covered the range from the 1970s up to the last 10 years, with references uniformly distributed across all the topics addressed. The only topic exclusively covered by sources from the 1990s is the Chance fracture [26, 107].

### Limb injuries

Lower extremity injuries are more prevalent, predominantly involving fractures of the femur, pelvis, and patella [76]. Frontal collisions occur when a vehicle abruptly decelerates upon colliding with another vehicle or a stationary object. Consequently, the impact forces are transmitted through the lower limbs of the front-seat occupants, affecting the knee-thigh-hip complex, with the hip joint being particularly susceptible to fractures or dislocations. Conversely, back seat occupants frequently exert force on the front seat using their extended upper limbs, with consequent fractures [9]. If children were secured using forward-facing child car seat systems, injuries below the knee, specifically affecting the tibia or fibula, are notably common. These lesions mainly result from collisions with the vehicle seatback positioned in front of the child’s seating area. Although these bone injuries are typical of frontal impacts, seatback interactions can occur in various other types of crashes [56].

Upper extremity injuries include fractures to the metacarpus, ulna, and radius [76]. These lesions occur in children seated in the front seat due to the action of the airbag compressing the region of the arms, neck, and face. For rear seat occupants, the extended upper limbs may be pushed against the front seat during the deceleration phase typical of frontal collisions [5, 37].

All articles discussed in this paragraph were published within the last 20 and 10 years, spanning from 2001 to 2017 [9, 37, 56, 76].

### Injury prevention strategies and technologies

In the last two decades, there has been increasing attention on the development of behavioural and technological prevention strategies to reduce the risk of death or severe injuries in children involved in motor vehicle accidents.

The primary injury prevention strategy highlighted in recent years is the use of child restraint systems. This is not limited to very young children, as studies have shown that children aged 2 through 6 years in child restraint systems experience a 21% reduction in mortality risk compared to those in seat belts alone. Child restraint systems offer significant safety advantages due to biomechanical considerations. They are designed to minimize the risk of ejection during a crash, distribute crash forces through stronger bones, limit crash forces by extending deceleration time, and potentially reduce contact with intruding vehicle structures. Child restraint systems also provide a better fit for restraints in children too small for adult-sized seat belts, offering a mechanical protection advantage [44].

Moreover, the positioning of child restraints on the seat has been observed to have an impact. In frontal motor vehicle crashes, pediatric models in rear-facing configurations generally show lower injury numbers than those in forward configurations, especially recommended up to at least 2 or 4 years of age. This is associated with a better support to the neck and the head, preventing rotational motion of the head [85, 91].

As children grow taller and older, they have a better chance of achieving a good seat belt fit. However, seat belt fit can be influenced by factors such as seat geometry and the locations of seat belt anchorages, independent of the occupant’s height and weight [98]. To optimize the transition from child restraint to seat belt use, the use of booster seats is recommended. Booster seats raise a child to the correct position, and data shows that children aged 6–8 using boosters are less likely to be injured compared to those using vehicle seat belts alone. Boosters adapt the child’s height to the vehicle restraint system, allowing comfortable knee bending and preventing a slouching sitting posture. Optimal protection is achieved when the pelvic bones are tightly coupled early and maintained throughout the event [44, 69, 85, 94].

Regarding seat position, it is strongly recommended to seat the child in the rear seat of the car. Even if the car is equipped with advanced front seat safety systems, including second-generation and modern airbags, it is recommended that all children aged 0–12 years should be seated in the rear rows. In cases of frontal or rear-end crashes, being in the front seat more than doubles the risk of fatality and being unrestrained increases fatality risk fourfold. For teenagers aged 13 and above, the reduction in injuries is not statistically significant, but it is suggested that young teenagers may benefit from rear seating similarly to their preteen counterparts [51, 60, 66].

To facilitate the storage and management of all this information, smartphone apps have been created to communicate child passenger safety. With the increasing interest in mobile health, the use of smartphone apps is considered rational for addressing health problems [84].

Injury prevention technologies have been devised to mitigate injuries in near-side impacts, which carry a high risk of severe or even fatal injuries. In these cases, the principal mechanism of injury is contact with the vehicle interior, mainly associated with intrusion into the occupant compartment at the child’s position, combined with lateral translation of the child’s body. The development of side airbags that protect the head and thorax reduces injury measurements, provided that the airbag properties are designed to consider these occupant sizes [72]. The use of side airbags limits the probability of serious head and trunk injuries, confining them to upper extremity fractures and concussions with brief loss of consciousness [66].

Moreover, air-bag technology has improved with the development of ‘smarter air bags’, with features that include sensors that can identify young passengers based on weight, and child restraint devices with on/off switches [7, 87].

All the articles discussed in this paragraph were uniformly published after the 2000s, with a clear division between those published before 2010 [44, 47, 51, 53, 57, 58, 60, 63, 66] and those published in the subsequent years, extending up to the most recent publications [69, 72, 84, 85, 88, 91, 94, 98].

### Forensic evaluation of injury patterns based on accident dynamics

The previous paragraphs have analyzed the primary injuries that may result from road traffic crashes involving children, along with an analysis of prevention strategies and technologies to mitigate them.

The evaluation of the temporal distribution of the various reports allows some considerations. First, articles assessing different injuries have been published throughout the whole timeframe covered by the review (1970–2023), with some peculiarities later discussed. On the other hand, papers on preventive measures are distributed in the last 20 years, in accordance with prevention awareness and the development of new technologies.

In-depth analysis of the temporal trends of head and neck injuries due to airbag direct impact has revealed a significant reduced reporting since the 2000s. This lower incidence can be explained as the application of the prevention strategy to place children in rear seats until their appropriate age.

On the other hand, head lesions associated with lateral impact and chest, abdominal and limb injuries are throughout reported along the entire period covered by the review. As a matter of fact, these lesions typically occur in high-speed road accidents, both lateral and frontal, in which the development of appropriate protective measures, as the integration of side airbags, remains a challenge in the field of engineering.

Analysis of injury patterns provides valuable information about accident dynamics and the type of crash in which children have been involved.

In motor vehicle accidents, child’s injuries can result from direct contact between the body and the internal components of the vehicle. This is particularly prominent in cases of vehicle rollovers and lateral impacts with other vehicles or stationary objects, such as poles and walls. On the other hand, acceleration and deceleration forces experienced in frontal and rear-end collisions can cause injuries through an indirect mechanism involving tissue stretching. In both dynamic scenarios, the primary cause of death is neurogenic shock. This can occur as a result of a direct impact to the head, leading to skull fractures, subdural haemorrhages, and cerebral contusions [3, 9, 59, 76, 92]. The indirect effects of acceleration-deceleration forces result in compressive and shear stresses on the brain and medulla, leading to subdural haemorrhages due to the rupture of meningeal bridging veins [9, 52, 59, 76, 80]. Although this dynamic is not directly related to skull fractures, it can be inferred from the presence of vertebral fractures [52, 80].

In the reported articles, children were correctly restrained in accordance with the specific requirements for their age. This suggests that the correct application of child protective equipment is not sufficient to prevent head injuries, especially in cases of high speed or multiple impacts. Moreover, the use of safety devices such as seat belts can also lead to injuries due to the direct impact of the belt on the body during a collision.

When using a lap-shoulder belt, injuries may include damage to neck and thoracic structures, resulting in sternum, rib, and clavicle fractures, as well as cardiac and aortic lesions [38, 97, 101, 104]. Alternatively, if the child is secured with a lap belt, this can lead to intra-abdominal injuries such as organ lacerations or perforations and aortic dissection [20, 24, 37, 93, 97]. The damages from the seat belt’s action are a result of the pressure exerted on the child’s body, in which muscle and bone structures are not fully developed yet. In such cases, an oblique or transverse abrasion corresponding to the seat belt position is typically observable during external examination.

However, during on-site inspections and the assessment of circumstantial data, it’s crucial to consider that specific injuries can also occur due to the improper use of child safety restraint systems.

The improper use of seat belts that do not correctly accommodate the infant’s body, leading to inadequate torso restraint, raises the risk of increased head movement and a higher likelihood of severe brain injury [8].

Improper placement of car restraints in the front seat can lead to injuries specifically related to airbag action during impact. In fact, standard guidelines for child restraints are based upon age and size. As a child ages, seating progresses to a forward-facing child safety seat to a belt-positioning booster seat with a three-point seat belt. In infants placed in forward-facing car seats, the deployment of airbags can result in facial compression, leading to facial bone and cervical spine fractures due to severe traumatic hyperextension [34, 39, 80]. External examinations may reveal facial thermal lesions or abrasions caused by contact with the airbag. Conversely, for infants positioned in rear-facing car seats, airbag deployment can lead to the child’s head being compressed between the restraint and the upright back of the passenger seat, resulting in skull fractures and brain contusions [32].

The forensic evaluation of injury patterns requires a comprehensive assessment of the child position in the vehicle at the time of the accident, in order to reconstruct the overall dynamics. The specific seat occupied by the child, the type of restraint used, and the adequacy of the restraint in relation to the child’s anthropometric characteristics should be carefully evaluated.

In a medicolegal context, a comprehensive assessment of the cause of injury resulting from a traffic crash requires expertise from multiple disciplines. Traffic crash reconstruction and engineering techniques are employed to evaluate the severity and direction of vehicle forces. Moreover, engineers test the proper functioning of protective devices such as child restraints, air bags, and seat belts. Medical-legal expertise is needed to understand the nature of collision-related injuries and their subsequent developments in non-fatal accidents. Biomechanical methods provide the connection between medically documented injuries and the reconstructed crash forces, and epidemiological approaches are used to properly classify and describe the crash and its outcomes in comparison to similar collisions. In litigation related to traffic accidents, the process of determining the degree to which safety restraint systems non-use contributed to the risk of injury or death is based on a comprehensive analysis of biomechanical, medical, and epidemiological factors [108].

Considering the complexity of the factors that can be involved in this road accident, it is crucial that there is a comprehensive exchange of information between the judge and the medical expert. This should include all relevant circumstantial elements, such as testimonies, accident videos, and kinematic engineering data related to car damage, skid marks, and proper use of safety devices.

## Conclusion

The review highlights that in motor vehicle accidents involving children, the forensic evaluation of both the cause of death and accident dynamics needs to consider several factors, such as the child’s age, the type of restraint system employed, and the specific passenger seat occupied. While brain damages remain the most frequently fatal across all age groups, certain specific injuries are linked to the restraint systems or airbag deployment.

When the accident dynamics are known, awareness of the distinct injury patterns can support forensic evaluation of sequence of events. Conversely, when the child location cannot be assumed, such as in case of multiple rollovers, the injuries patterns can provide useful information for the events reconstruction.

## Data Availability

The data presented in this study are available on request from the corresponding author.
